# Survival in patients with intermediate or high grade non-Hodgkin's lymphoma: meta-analysis of randomized studies comparing third generation regimens with CHOP

**DOI:** 10.1054/bjoc.2000.1566

**Published:** 2001-02

**Authors:** A Messori, M Vaiani, S Trippoli, L Rigacci, M Jerkeman, G Longo

**Affiliations:** Drug Information Center Azienda Ospedaliera Careggi, Firenze, Italy; Department of Haematology, Azienda Ospedaliera Careggi, Firenze, Italy; Department of Oncology, Jubileum Institute, Lund University Hospital, Sweden

**Keywords:** non-Hodgkin's lymphoma, survival, meta-analysis, chemotherapy

## Abstract

In patients with intermediate or high grade non-Hodgkin lymphoma (NHL), third generation chemotherapy regimens have been introduced to improve survival in comparison with the standard CHOP regimen. However, most studies have found no difference between these two treatments. We conducted a meta-analysis to assess the effectiveness of third generation regimens as compared with CHOP. Our study included the randomized controlled trials published in English from 1970 to 1999. After a Medline search, 5 trials were found to meet our inclusion criteria. A total of 1982 patients, that were enrolled in these trials, were included in the survival meta-analysis. Our methodology retrieved patient-level information from all of these subjects; survival up to 9 years after randomization was compared between the two treatment options. The results of our meta-analysis showed that, in comparison with CHOP, third generation chemotherapy did not prolong survival at levels of statistical significance (chi-square by log-rank test = 1.44, *P* = 0.23). The relative death risk for third generation regimens vs. CHOP was 0.92 (95%CI: 0.80 to 1.06;*P * = 0.26). We conclude that, on the basis of our meta-analysis, third generation regimens do not confer any survival benefit to patients with intermediate or high grade NHL as compared with CHOP. © 2001 Cancer Research Campaign http://www.bjcancer.com


*British Journal of Cancer* (2001) **84**(3), 303–307
© 2001 Cancer Research Campaign
doi: 10.1054/ bjoc.2000.1566, available online at http://www.idealibrary.com on IDEAL®

http://www.bjcancer.com

### Survival in patients with intermediate or high grade non-Hodgkin's lymphoma: meta-analysis of randomized studies comparing third generation regimens with CHOP

**A Messori[1], M Vaiani[1], S Trippoli[1], L Rigacci[2], M Jerkeman[3] and G Longo[2]**

[1]Drug Information Center Azienda Ospedaliera Careggi, Firenze, Italy; [2]Department of Haematology, Azienda Ospedaliera Careggi, Firenze, Italy; [3]Department of Oncology, Jubileum Institute, Lund University Hospital, Sweden

**Summary** In patients with intermediate or high grade non-Hodgkin lymphoma (NHL), third generation chemotherapy regimens have been introduced to improve survival in comparison with the standard CHOP regimen. However, most studies have found no difference between these two treatments. We conducted a meta-analysis to assess the effectiveness of third generation regimens as compared with CHOP. Our study included the randomized controlled trials published in English from 1970 to 1999. After a Medline search, 5 trials were found to meet our inclusion criteria. A total of 1982 patients, that were enrolled in these trials, were included in the survival meta-analysis. Our methodology retrieved patient-level information from all of these subjects; survival up to 9 years after randomization was compared between the two treatment options. The results of our meta-analysis showed that, in comparison with CHOP, third generation chemotherapy did not prolong survival at levels of statistical significance (chi-square by log-rank test = 1.44, $P = 0.23$). The relative death risk for third generation regimens vs. CHOP was 0.92 (95%CI: 0.80 to 1.06; $P = 0.26$). We conclude that, on the basis of our meta-analysis, third generation regimens do not confer any survival benefit to patients with intermediate or high grade NHL as compared with CHOP. © 2001 Cancer Research Campaign http://www.bjcancer.com

**Keywords**: non-Hodgkin's lymphoma; survival; meta-analysis; chemotherapy

During the last two decades, the CHOP regimen is considered to be the standard treatment for intermediate or high-grade non-Hodgkin lymphoma (NHL). In recent years, however, several schemes of more aggressive chemotherapy (e.g. the so-called third generation regimens) have been devised and tested both in controlled and uncontrolled clinical studies. These schemes of aggressive chemotherapy have been reported to increase the response rate on the short-term, but have an uncertain impact on long-term survival (Martelli et al, 1997).

There are numerous Phase II studies reporting the results with third generation chemotherapy, but these do not permit to define the therapeutic role of these new regimens in comparison with CHOP. On the other hand, the results of Phase-III randomized trials evaluating third generation schemes vs. CHOP (Gordon et al, 1992; Fisher et al, 1993; Cooper et al, 1994; Montserrat et al, 1996; Wolf et al, 1997; Jerkeman et al, 1999) have never been included in a systematic overview or in a meta-analysis.

In the present study, we conducted a meta-analysis of the survival data obtained in randomized controlled trials (RCTs) comparing third generation regimes with CHOP.

### METHODS

### Study design

The aim of our study was to evaluate survival for the two following therapeutic options for patients with intermediate or high grade NHL: a) third generation regimens (namely MACOP-B or m-BACOD or ProMace-CytaBOM or any other regimen which the author of the trial originally defined as third generation); b) CHOP. Our analysis consisted of two sequential phases: 1) Literature search of the RCTs that evaluated survival for these two therapeutic options; 2) Survival analysis with meta-analytic pooling of the results from the pertinent trials and with statistical testing.

Our survival meta-analysis was carried out through the following procedure. Firstly, the patient-level information on survival was retrieved from the cohorts enrolled in the various studies; subsequently, the survival difference between third generation regimens and CHOP was assessed by pooling the individual data of survival across the pertinent studies and by constructing the two survival curves for third generation regimens and CHOP.

### Literature search

This part of our study included:

- a MEDLINE search on the Internet (WWW Entrez, PubMed Data Base, Internet address: 'http://www4.ncbi.nlm.nih.gov/PubMed/', search from 1 January 1970 to 29 February 2000, keywords: 'non-Hodgkin', 'survival' and 'randomized' or 'randomised');
- a search on the IDIS compact-disk (Iowa Drug Information System, Iowa City, USA; computer search from January 1985 to December 1999; keywords: 'non-Hodgkin', 'survival' and 'randomized' or 'randomised');
- consultation of reviews, textbooks and experts in this particular field of study.

*Received 12 May 2000*
*Revised 13 September 2000*
*Accepted 11 October 2000*

*Correspondence to:* A Messori

In addition, we reviewed all the references listed in the trials we found. Only the trials published in English were considered.

### Meta-analysis of survival data

The studies identified by our literature search were included in the meta-analysis when they met the following criteria: a) enrolment of patients with intermediate or high-grade NHL; b) randomized design; c) treatment assignment to a third generation regimen (treatment group receiving either MACOP-B or m-BACOD or ProMACE-CytaBOM or any other regimen defined as third generation) or CHOP (control group); c) survival assessment (with presentation of the survival graph).

Our survival meta-analysis was carried out using individual patient information (Stewart and Parmar, 1993; Jeng et al, 1995; Oxman et al, 1995; Steinberg et al, 1997), i.e. survival length and status at the last contact. In particular, the data of individual survival were derived either from the original raw data provided by the trial's authors (who were contacted for this purpose) or from the information contained in the figures that had originally reported the survival graphs for these patients.

After obtaining these survival data for all subjects enrolled in the pertinent studies, our analysis generated a pooled survival curve for third generation regimens and a pooled survival curve for CHOP. In the survival comparison between the two treatments, standard life-table methods (Kaplan-Meier analysis) and standard techniques for univariate (log-rank test) or multivariate testing (Cox model for multivariate relative risk estimation) were used. When possible, the survival data were analysed using an intention-to-treat approach. To construct the meta-analysis plot, crude death rates from the individual studies (with their respective odds-ratios) were pooled according to the grand-total method of Collins et al (1985); in this way, the summary (or meta-analytic) odds-ratio of death for the comparison between third generation regimens and CHOP was estimated and presented in graphical form.

In a secondary analysis, the meta-analytic comparison between third generation regimens and CHOP was re-assessed using trial-specific aggregate survival data, and so without constructing patient-level information. The statistical method utilized for this secondary analysis has been described previously (Messori and Rampazzo, 1993) and reflects a traditional approach for conducting a survival meta-analysis with no access to individual patient data. Its application produced a meta-analytic odds-ratio of death for third generation regimens vs. CHOP.

### RESULTS

### Clinical material

5 trials (Table 1) met the inclusion criteria of our meta-analysis. The total number of patients enrolled in these 5 trials was 1203 for third generation regimens and 779 for CHOP. The crude survival rates were 528/1203 (44%) for third generation regimens and 366/779 (47%) for CHOP. The third generation regimens used in these trials included MACOP-B ($n$ = 524; 44%), m-BACOD ($n$ = 374; 31%), and ProMACE-CytaBOM ($n$ = 305; 25%). The study by Linch et al (1996) was excluded because the dose scheduling of the CHOP regimen in the control group differed from the traditional 3-week administration (this study found no difference between the third-generation regimen and CHOP). The scheduling and dose intensity for the CHOP group was very similar across the 5 studies included in our analysis.

The survival information for these patients was derived from: a) Figure 2 for the study by Wolf et al (1997) (survival graphs based on the intention-to-treat approach); b) Figure 2 for the study by Fisher et al (1993) (survival graphs based on the intention-to-treat approach); c) Figure 1 for the study by Gordon et al (1992) (by-treatment analysis with no survival graph based on the intention-to-treat approach); d) Figure 4 for the study by Montserrat et al (1996) (survival graphs based on the intention-to-treat approach); e) the original raw data of survival in the case of the study by Jerkeman et al (1999) (data based on the intention-to-treat approach excluding those patients who were randomized in the absence of the inclusion criteria).

In the 2 trials by Gordon et al (1992) and Wolf et al (1997), the legends of the survival graphs provided complete information on the time distribution of deaths and on the time distribution of right-censored patients. In the 2 trials by Fisher et al (1993) and by Montserrat et al (1996), the survival information was estimated by the approximate procedure described in Appendix 1. The individual survival times of the 1982 patients are not presented herein, but have been published on the Internet site http://members.nbci.com/sifotpn/supplements/NHL.htm/labsifo/supplements/nh13g.htm.

### Survival meta-analysis

Our survival meta-analysis yielded the two survival curves shown in Figure 1. The survival rates (± standard error) for the third-generation group were at 55.7% (± 1.5%) at 36 months, 53.7% (± 1.6%) at 48 months, 51.2% (± 1.6%) at 60 months, 49.1% (± 1.7%) at 72 months; those for the CHOP group were 57.1% (± 1.9%) at 36 months, 53.3% (± 1.9%) at 48 months, 45.8% (± 2.1%) at 60 months, 45.1% (± 2.1%) at 72 months. After 72 months, the number of patients at risk becomes relatively small, and so the two curves are less informative.

The survival difference between the two treatments was not significant (chi-square by log-rank test with 1 df = 1.44, $P$ = 0.23). The Cox analysis (that considered the effect on survival of two variables: 'study', introduced as a categorical variable stratified on 5 levels, and 'treatment' introduced as a categorical variable stratified on 2 levels) calculated a relative death risk of 0.92 for third generation regimens vs. CHOP (95% CI: 0.80 to 1.06; $P$ = 0.26). The study-specific values of relative death risk (Cox model) were not significantly different from one another (study by Wolf et al (1997): 1.00 with 95% CI of 0.86 to 1.17, $P$ = 0.99; study by Fisher et al (1993): 1.02 with 95% CI of 0.91 to 1.14, $P$ = 0.76; study by Montserrat et al (1996): 1.08 with 95% CI of 0.99 to 1.30, $P$ = 0.42; study by Jerkeman et al (1999): 0.91 with 95% CI of 0.78 to 1.05, $P$ = 0.20; all risk values calculated in comparison with the study by Gordon et al (1992) which was assumed to have death risk = 1); these data show that the inter-trial heterogeneity of the clinical material was acceptable. The meta-analysis plot based on crude death rates is shown in Figure 2.

In the meta-analysis based on aggregate survival data, the meta-analytic odds-ratio of death for third generation regimens vs. CHOP was 0.88 at 60 months (95% CI: 0.75 to 1.04; $P$ = 0.15), which was very close to the relative risk obtained from the meta-analysis of individual patient data (0.92 with 95% CI of 0.80 to 1.06).

### DISCUSSION

In our meta-analysis, the survival pattern for third generation regimens was not significantly different from that of CHOP (Figure 1),

© 2001 Cancer Research Campaign

**Table 1** Patients included in the treatment group (third generation chemotherapy) and in the control group (CHOP) of the 5 RCTs

| Study | Inclusion criteria | Follow-up length (y) | Treatment group | | Control group | | Survival comparison between third generation regimens vs. CHOP | Statistical level for the survival comparison |
|---|---|---|---|---|---|---|---|---|
| | | | No. of patients | Type of chemotherapy | No. of patients | Type of chemotherapy* | | |
| Cooper et al (1994) and Wolf et al (1997) | Stage I–IV disease; intermediate or high grade disorder; age greater than 16 years | 9 | 125 | MACOP-B | 111 | CHOP | Crude rate of 63/125 for MACOP-B vs. 68/111 for CHOP; 5-y rate of 54% for MACOP-B vs. 41% for CHOP | $P = 0.035$ |
| Fisher et al (1993) | Stage II–IV disease; intermediate or high grade disorder; no age restrictions | 5 | 674 | MACOP-B ($n = 218$§) or m-BACOD ($n = 223$ §) or ProMACE-CytaBOM ($n = 233$ §) | 225 | CHOP | Crude death rate of 283/674 for third generation vs. 88/225 for CHOP; 3-y rate of 50% to 52% for third generation vs. 54% for CHOP | $P = 0.90$ |
| Jerkeman et al (1999) | Stage II–IV disease; high grade disorder; age between 18 and 67 years | 8 | 181 | MACOP-B | 193 | CHOP | 5-yr rate of 60% for MACOP-B vs. 59% for CHOP | $P$ = NS |
| Gordon et al (1992) | Stage III–IV disease; high grade disorder; no age restrictions | 6 | 151 | m-BACOD | 174 | CHOP | Crude death rate of 71/151 for m-BACOD vs. 91/174 for CHOP (by-treatment approach) or 90/193 for m-BACOD vs. 102/199 for CHOP (intention-to-treat approach); 5-y rate of 49% for m-BACOD vs. 48% for CHOP | $P = 0.489$ (by-treatment approach) or $P = 0.50$ (intention-to-treat approach) |
| Montserrat et al (1996) | Stage II–IV disease; intermediate or high grade disorder; no age restrictions | 6 | 72 | ProMACE-CytaBOM | 76 | CHOP | Crude death rate of 40/72 for third generation vs. 38/76 for CHOP; 5-y rate of 42% in both groups | $P$ = NS |

§ In our analysis, these 3 different third-generation schemes were pooled into a single treatment group ($n = 674$) which was compared to the control group (225 patients given CHOP). *The two studies by Gordon et al and Jerkeman et al administered at least 8 cycles of CHOP in responders, while the two studies of Montserrat et al and Cooper et al administered at least 6 cycles (together with the criterion of 2 cycles after complete response for the study of Cooper); Fisher et al administered 8 cycles of CHOP (unless progressive disease developed). NS = not significant.

and the $P$ values for this comparison ($P = 0.23$ and $P = 0.26$ in the two analyses of individual patient data) remained very far from the conventional level of statistical significance ($P = 0.05$). Hence, the main conclusion resulting from our analysis is that third generation regimens do not confer any survival benefit to NHL patients. The results of our inter-study comparison based on the Cox model showed that the heterogeneity across the 5 trials was not statistically significant; this finding therefore supports the reliability of our meta-analytical calculations. Among the 5 trials included in our analysis (Table 1), there were 4 negative studies (Gordon et al, 1992; Fisher et al, 1993; Montserrat et al, 1996; Jerkeman et al, 1999) together with a single positive study (Wolf et al, 1997) that found a survival improvement. The positive study has very similar characteristics in comparison with the others in terms of both patient selection criteria (very similar to the studies by Fisher et al (1993) and Montserrat et al (1996)) and type of aggressive chemotherapy (identical to the studies by Fisher et al (1993) and Jerkeman et al (1999)).

Since the 5 clinical trials examined in our study (Gordon et al, 1992; Fisher et al, 1993; Montserrat et al, 1996; Jerkeman et al, 1999; Wolf et al, 1997) do not show any significant advantage for third generation chemotherapy in comparison with CHOP, our results do not support the choice of third generation regimens as the treatment for the control group that has been made in randomized studies testing new therapeutic approaches for NHL (e.g. high-dose chemotherapy with haematopoietic stem cell rescue vs.

*© 2001 Cancer Research Campaign* 

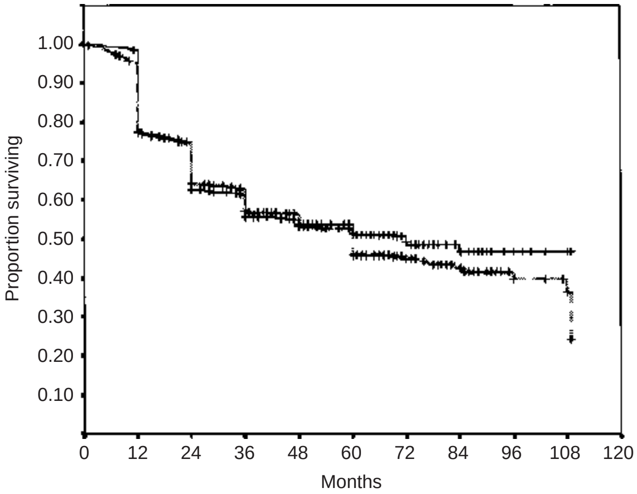

**Figure 1** Survival in patients with intermediate or high-grade NHL: the solid curve shows the survival pattern for patients treated with third generation regimens (*n* = 1203), while the dashed curve refers to patients treated with CHOP (*n* = 779). Both curves were calculated by standard life-table methods using individual patient data. In the third-generation group, the number of patients at risk was 590 at 24 months, 386 at 48 months, 111 at 72 months, and 12 at 96 months; the same figures for the CHOP group were 377, 240, 83 and 13, respectively. See text for details

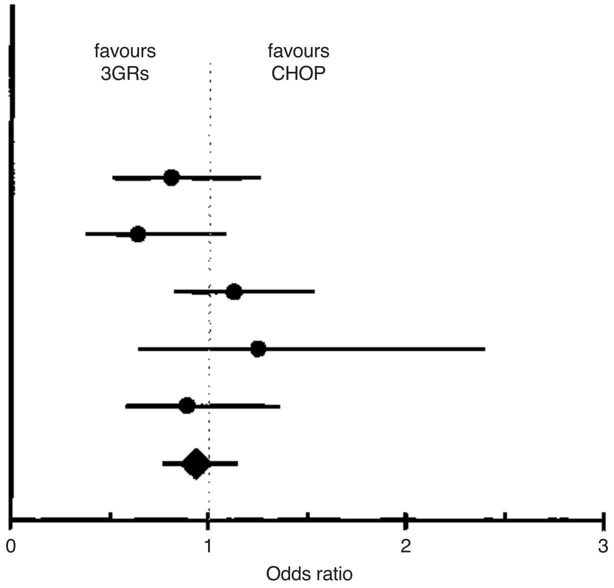

**Figure 2** Comparison of crude death rates between third-generation regimens and CHOP: values of study-specific odds-ratio (circles) with 95% CIs and summary odds-ratio (diamond) with 95% CI. The vertical dotted line represents identity in death rate between the two patient groups. From top to bottom, the first 5 data sets show the study-specific odds-ratio for the trial of Gordon et al, Wolf et al, Fisher et al, Montserrat et al and Jerkeman et al, respectively; the sixth data set shows the results of our meta-analysis (summary odds-ratio). The two graph sections that favour 3GRs (left) or CHOP (right) are presented according to the standard scheme of meta-analysis graphs. Abbreviations: 3GRs = third generation regimens a third-generation regimen (Gianni et al, 1997) or comparison of two third-generation regimens with one another (Mazza et al, 1995; Guglielmi et al, 1989)). Nonetheless, the fact that the control group of these trials received third generation regimens (instead of CHOP), discloses a quite widespread, though unproven, belief that these regimens are more effective, at least in certain subsets of NHL patients (e.g. young subjects who are thought to better tolerate the full doses of third generation regimens). Although one (Wolf et al, 1997) of the 5 clinical studies found that the survival advantage resulting from third generation regimens was restricted to the subset of younger patients (and was instead much smaller in older subjects), the other 4 studies did not confirm this finding or did not specifically address this hypothesis. Hence, this question remains open and cannot be settled by the results of our analysis.

In comparing third-generation regimens with CHOP, our analysis showed that the relative death risk was 0.92 and that the 95% CI for this relative risk ranged from 0.80 to 1.06. Hence, our findings are compatible (at the 5% level) with the hypothesis that third-generation regimens are 20% better than CHOP in relative terms, but are also compatible with the hypothesis that CHOP is 6% better than third-generation regimens. If new studies will be designed to test again the hypothesis that third-generation regimens improve survival (according to our data, the survival improvement is, in absolute terms, from 45.1% to 49.1% at 72 months with a relative difference of +8%), their sample size should be of at least 2360 patients for the third-generation regimen group and 2360 patients for the CHOP group (statistical power calculations made using the method of Edmiston et al (1993) with alpha = 0.10 (two-tailed) and (1-beta) = 0.80). If these studies are aimed at detecting a relative survival improvement of +10%, +15% or +20%, the suggested sample size for each of the two study arms reduces to 1514 patients, 664 patients or 379 patients, respectively. In the light of these statistical power calculations, planning new controlled studies on this issue will require a patient population of this size, but one could wonder whether such experimental effort is worthwhile. In any case, new studies based on small patient populations would make little sense because they would be bound to generate no useful results. Fisher et al (1993) have shown that the cost of third generation regimens can vary considerably, but is always much higher than that of CHOP. According to Fisher, if the cost of the drugs used in a planned course of CHOP is assigned a value of 1.00, the cost of MACOP-B is 1.13, that of ProMACE-CytaBOM 1.44, and that of m-BACOD is 2.26 (on the basis of average wholesale prices of US in 1993). A more complete economic analysis would imply the assessment of the costs of hospitalization and day hospital, which are known to be much higher for third-generation regimens than for CHOP, and so this would greatly enhance the cost difference between the two treatments. While a specific cost-effectiveness calculation would require a separate study, this preliminary information on costs and clinical benefits favours CHOP with a quite clear indication due to a lower cost per patient and similar therapeutic efficacy in comparison with third-generation regimens.

The main difference between CHOP and the third-generation regimens is the number of chemotherapeutic drugs. In m-BACOD and MACOP-B, methotrexate and bleomycin was added to the drugs in CHOP. In addition, the ProMACE-CytaBOM regimen included etoposide and cytarabine. The rationale was to overcome chemotherapy resistance and improve curability by addition of non-crossresistant drugs, in line with the hypothesis by Goldie et al (1982). However, to avoid excess toxicity, the dose intensity (DI) of cyclophosphamide and doxorubicin had to be reduced in the newer regimens, with the exception of a slightly higher DI of doxorubicin in MACOP-B.

© 2001 Cancer Research Campaign

In light of the present analysis, one may conclude that addition of bleomycin and methotrexate is insufficient to overcome chemotherapy resistance, and that the drugs included in the CHOP regimen (cyclophosphamide, doxorubicin and vincristine) are more important for therapeutic efficacy. In the design of future studies, if one accepts the view that further testing of third-generation based on very large-scale studies is not worthwhile, alternative approaches should be sought, such as further escalation of doxorubicin and cyclophosphamide.

### APPENDIX 1: APPROXIMATIONS INTRODUCED IN OUR SURVIVAL ANALYSIS

In our analysis of the trials by Fisher et al (1993) and Montserrat et al (1996), each of the two survival curves (treatment group and controls) was analysed by the approximate method described by Fine et al (1993) in order to convert the aggregate survival data that had originally been published in graphical form into values of individual survival. This method determines the distribution over time of deaths and of terminations of follow-up (i.e. cases of right 'censored patients') using a graphical analysis of the published curves. The calculation requires also the knowledge of the total number of patients and the total number of deaths (reported separately for the two arms of the study under examination), which were both directly presented in the text of the two articles.

This approximated method for constructing individual survival times has often been used in previous retrospective overviews and in meta-analyses of survival data (Fine et al, 1993; Messori et al, 1994; Bardelli et al, 1995; Trallori et al, 1995; Ferradina et al, 1997; Messori et al, 1999a, 1999b). The computer programme implementing this method has been recently published (Messori et al, 2000).

### REFERENCES

Bardelli F, Messori A, Rampazzo R, Alberti A and Martini N (1995) Effect of recombinant or lymphoblastoid alpha interferon on ALT in patients with chronic hepatitis C or chronic non-A non-B hepatitis: a meta-analysis. *Clin Drug Invest* **9**: 239–254

Collins R, Yusuf S and Peto R (1985) Overview of randomised trials of diuretics in pregnancy. *BMJ* **290**: 17–23

Cooper IA, Wolf MM, Robertson TI, Fox RM, Matthews JP, Stone JM, Chong Ding J, Dart G, Matthews J, Firkin FC, Lowenthal RM and Ironside P for the Australian and New Zealand Lymphoma Group (1994) Randomized comparison of MACOP-B with CHOP in patients with intermediate-grade non-Hodgkin's lymphoma. *J Clin Oncol* **12**: 769–778

Edmiston CE, Josephson A, Pottinger J, Ciacco-Tsivitis M and Palenik C (1993) The numbers game: sample-size determination. *Am J Infect Control* **3**: 151–154

Ferrandina G, Scambia G, Bardelli F, Benedetti Panici P, Mancuso S and Messori A (1997) Relationship between cathepsin-D content and disease-free survival in node-negative breast cancer patients: a meta-analysis. *Br J Cancer* **76**: 661–666

Fine HA, Dear KBG, Loeffler JS, Black PML and Canellos GP (1993) Meta-analysis of radiation therapy with and without adjuvant chemotherapy for malignant gliomas in adults. *Cancer* **71**: 2585–2592

Fisher RI, Gaynor ER, Dahlberg S, Oken MM, Grogan TM, Mize EM, Glick JH, Coltman CA and Miller TP (1993) Comparison of a standard regimen (CHOP) with three intensive chemotherapy regimens for advanced non-Hodgkin's lymphoma. *N Engl J Med* **328**: 1002–1006

Gianni AM, Bregni M, Siena S, Brambilla C, Di Nicola M, Lombardi F, Gandola L, Tarella C, Pileri A, Ravagnani F, Valagussa P and Bonadonna G (1997) High-dose chemotherapy and autologous bone marrow transplantation compared with MACOP-B in aggressive B-cell lymphoma. *N Engl J Med* **336**: 1290–1297

Goldie JH, Coldman AJ and Gudauskas GA (1982) Rationale for the use of alternating non-cross-resistant chemotherapy. *Cancer Treat Rep* **66**: 439–449

Gordon LI, Harrington D, Andersen J, Colgan J, Glick J, Neiman R, Mann R, Resnick GD, Barcos M, Gottlieb A and O'Connell M (1992) Comparison of a second-generation combination chemotherapeutic regimen (m-BACOD) with a standard regimen (CHOP) for advanced diffuse non-Hodgkin's lymphoma. *N Engl J Med* **327**: 1342–1349

Guglielmi C, Gherlinzoni F, Amadori S, Mazza P, Mantovani L, Lauria F, Martelli M, Zinzani PL, Greco V and Poletti G (1989) A phase III comparative trial of m-BACOD vs m-BNCOD in the treatment of stage II–IV diffuse non-Hodgkin's lymphomas. *Haematologica* **74**: 563–569

Jeng GT, Scott JR and Burmeister LF (1995) A comparison of meta-analytic results using literature vs individual patient data: paternal cell immunization for recurrent miscarriage *JAMA* **274**: 830–836

Jerkeman M, Anderson H, Cavallin-Stahl E, Dictor M, Hagberg H, Johnson A, Kaasa S, Kvaloy S, Sundström C and Åkerman M for the Nordic Lymphoma Group (1999). CHOP versus MACOP-B in aggressive lymphoma – a Nordic Lymphoma Group randomised trial. *Ann Oncol* **10**: 1079–1086

Linch DC, Vaughan Hudson B, Hancock BW, Hoskin PJ, Cunningham DC, Newland AC, Milligan DW, Stevenson PA, Wood JK, MacLennan KA, Anderson L, Gregory WM, Vaughan and Hudson G on behalf of the British National Lymphoma Investigation (1996). A randomised comparison of a third generation regimen (PACEBOM) with a standard regimen (CHOP) in patients with histologically aggressive non-Hodgkin's lymphoma: a British National Lymphoma Investigation report. *Br J Cancer* **74**: 318–322

Martelli M, De Sanctis Vitaliana, Avvisati G and Mandelli F (1997). Current guidelines for the management of aggressive non-Hodgkin's lymphoma. *Drugs* **53**: 957–972

Mazza P, Zinzani PL and Martelli M et al (1995) MACOP-B vs. F-MACHOP regimen in the treatment of high-grade non-Hodgkin's lymphomas. *Leuk Lymphoma* **16**: 457–463

Messori A and Rampazzo R (1993) Meta-analysis of clinical trials based on censored end-points: simplified theory and implementation of the statistical algorithms on a microcomputer. *Comput Progr Meth Biomed* **40**: 261–267

Messori A, Brignola C, Trallori G, Rampazzo R, Bardazzi G, Belloli C, d'Albasio G, De Simone G and Martini N (1994) Effectiveness of 5-aminosalicylic acid (5-ASA) for maintaining remission in patients with Crohn's disease: a meta-analysis. *Am J Gastroenterol* **89**: 692–698

Messori A, Bosi A, Bacci S, Laszlo D, Trippoli S, Locatelli F, Van Lint MT, Di Bartolomeo P and Amici A on behalf of the GITMO (1999a). Retrospective survival analysis and cost-effectiveness evaluation of second allogeneic bone marrow transplantation in patients with acute leukemia. *Bone Marrow Transplant* **23**: 489–495

Messori A, Trippoli S, Becagli P and Zaccara G (1999b). Cost-effectiveness of riluzole in amyotrophic lateral sclerosis. *Pharmacoeconomics* **16**: 153–163

Messori A, Trippoli G, Vaiani M and Cattel F (2000) Survival meta-analysis of individual patient data and survival meta-analysis of published (aggregate) data. *Clin Drug Invest* **20**: 309–316.

Montserrat E, Vinolas N, Lopez-Guillermo A, Hernandez-Nieto L, Zubizarreta A, Maldonado J, Alcala A, Faura MV, Llorente A, Bladé J, Fontanillas M and Estapé (1996) CHOP vs. ProMACE-CytaBOM in the treatment of aggressive non-Hodgkin's lymphomas: long-term results of a multicenter randomized trial. *Eur J Haematol* **57**: 377–383

Oxman AD, Clarke MJ and Stewart LA (1995). From science to practice: meta-analysis using individual patient data are needed. *JAMA* **274**: 845–846

Steinberg KK, Smith SJ, Stroup DF, Olkin I, Lee NC, Williamson GD and Thacker SB (1997) Comparison of effect estimates from a meta-analysis of summary data from published studies and from a meta-analysis using individual patient data for ovarian cancer studies. *Am J Epidemiol* 15; **145**: 917–925

Stewart LA and Parmar MK (1993) Meta-analysis of the literature or of individual patient data: is there a difference? *Lancet* **341**: 418–422

Trallori G, Messori A, Scuffi C, Bardazzi G, Silvano R, d'Albasio G and Pacini F (1995) Effectiveness of 5-aminosalicylic acid enemas for maintaining remission in patients with left-sided ulcerative colitis: a meta- and economic analysis. *J Clin Gastroenterol* **20**: 257–259

Wolf M, Matthews JP, Stone J, Cooper IA, Robertson TI and Fox RM for the Australian and New Zealand Lymphoma Group (1997) Long-term survival advantage of MACOP-B over CHOP in intermediate-grade non-Hodgkin's lymphoma. *Ann Oncol* 8 (Suppl.1): S71–S75

*© 2001 Cancer Research Campaign*

## References

[PDF_00418] Collins R., Yusuf S., Peto R. (1985). Overview of randomised trials of diuretics in pregnancy.. Br Med J (Clin Res Ed).

[PDF_00420] Cooper I. A., Wolf M. M., Robertson T. I., Fox R. M., Matthews J. P., Stone J. M., Ding J. C., Dart G., Matthews J., Firkin F. C. (1994). Randomized comparison of MACOP-B with CHOP in patients with intermediate-grade non-Hodgkin's lymphoma. The Australian and New Zealand Lymphoma Group.. J Clin Oncol.

[PDF_00425] Edmiston C. E., Josephson A., Pottinger J., Ciacco-Tsivitis M., Palenik C. (1993). The numbers game: sample-size determination.. Am J Infect Control.

[PDF_00427] Ferrandina G., Scambia G., Bardelli F., Benedetti Panici P., Mancuso S., Messori A. (1997). Relationship between cathepsin-D content and disease-free survival in node-negative breast cancer patients: a meta-analysis.. Br J Cancer.

[PDF_00430] Fine H. A., Dear K. B., Loeffler J. S., Black P. M., Canellos G. P. (1993). Meta-analysis of radiation therapy with and without adjuvant chemotherapy for malignant gliomas in adults.. Cancer.

[PDF_00433] Fisher R. I., Gaynor E. R., Dahlberg S., Oken M. M., Grogan T. M., Mize E. M., Glick J. H., Coltman C. A., Miller T. P. (1993). Comparison of a standard regimen (CHOP) with three intensive chemotherapy regimens for advanced non-Hodgkin's lymphoma.. N Engl J Med.

[PDF_00437] Gianni A. M., Bregni M., Siena S., Brambilla C., Di Nicola M., Lombardi F., Gandola L., Tarella C., Pileri A., Ravagnani F. (1997). High-dose chemotherapy and autologous bone marrow transplantation compared with MACOP-B in aggressive B-cell lymphoma.. N Engl J Med.

[PDF_00442] Goldie J. H., Coldman A. J., Gudauskas G. A. (1982). Rationale for the use of alternating non-cross-resistant chemotherapy.. Cancer Treat Rep.

[PDF_00445] Gordon L. I., Harrington D., Andersen J., Colgan J., Glick J., Neiman R., Mann R., Resnick G. D., Barcos M., Gottlieb A. (1992). Comparison of a second-generation combination chemotherapeutic regimen (m-BACOD) with a standard regimen (CHOP) for advanced diffuse non-Hodgkin's lymphoma.. N Engl J Med.

[PDF_00450] Guglielmi C., Gherlinzoni F., Amadori S., Mazza P., Mantovani L., Lauria F., Martelli M., Zinzani P. L., Greco V., Poletti G. (1989). A phase III comparative trial of m-BACOD vs m-BNCOD in the treatment of stage II-IV diffuse non-Hodgkin's lymphomas.. Haematologica.

[PDF_00454] Jeng G. T., Scott J. R., Burmeister L. F. (1995). A comparison of meta-analytic results using literature vs individual patient data. Paternal cell immunization for recurrent miscarriage.. JAMA.

[PDF_00457] Jerkeman M., Anderson H., Cavallin-Ståhl E., Dictor M., Hagberg H., Johnson A., Kaasa S., Kvaløy S., Sundström C., Akerman M. (1999). CHOP versus MACOP-B in aggressive lymphoma--a Nordic Lymphoma Group randomised trial.. Ann Oncol.

[PDF_00461] Linch D. C., Vaughan Hudson B., Hancock B. W., Hoskin P. J., Cunningham D. C., Newland A. C., Milligan D. W., Stevenson P. A., Wood J. K., MacLennan K. A. (1996). A randomised comparison of a third-generation regimen (PACEBOM) with a standard regimen (CHOP) in patients with histologically aggressive non-Hodgkin's lymphoma: a British National Lymphoma Investigation report.. Br J Cancer.

[PDF_00468] Martelli M., De Sanctis V., Avvisati G., Mandelli F. (1997). Current guidelines for the management of aggressive non-Hodgkin's lymphoma.. Drugs.

[PDF_00471] Mazza P., Zinzani P. L., Martelli M., Fiacchini M., Bocchia M., Pileri S., Falini B., Martelli M. F., Amadori S., Papa G. (1995). MACOP-B vs F-MACHOP regimen in the treatment of high-grade non-Hodgkin's lymphomas.. Leuk Lymphoma.

[PDF_00481] Messori A., Bosi A., Bacci S., Laszlo D., Trippoli S., Locatelli F., Van Lint M. T., Di Bartolomeo P., Amici A. (1999). Retrospective survival analysis and cost-effectiveness evaluation of second allogeneic bone marrow transplantation in patients with acute leukemia. Gruppo Italiano Trapianto di Midollo Osseo.. Bone Marrow Transplant.

[PDF_00477] Messori A., Brignola C., Trallori G., Rampazzo R., Bardazzi G., Belloli C., d'Albasio G., De Simone G., Martini N. (1994). Effectiveness of 5-aminosalicylic acid for maintaining remission in patients with Crohn's disease: a meta-analysis.. Am J Gastroenterol.

[PDF_00474] Messori A., Rampazzo R. (1993). Meta-analysis of clinical trials based on censored end-points: simplified theory and implementation of the statistical algorithms on a microcomputer.. Comput Methods Programs Biomed.

[PDF_00486] Messori A., Trippoli S., Becagli P., Zaccara G. (1999). Cost effectiveness of riluzole in amyotrophic lateral sclerosis. Italian Cooperative Group for the Study of Meta-Analysis and the Osservatorio SIFO sui Farmaci.. Pharmacoeconomics.

[PDF_00492] Montserrat E., García-Conde J., Viñolas N., López-Guillermo A., Hernández-Nieto L., Zubizarreta A., Maldonado J., Alcalá A., Faura M. V., Llorente A. (1996). CHOP vs. ProMACE-CytaBOM in the treatment of aggressive non-Hodgkin's lymphomas: long-term results of a multicenter randomized trial.(PETHEMA: Spanish Cooperative Group for the Study of Hematological Malignancies Treatment, Spanish Society of Hematology).. Eur J Haematol.

[PDF_00497] Oxman A. D., Clarke M. J., Stewart L. A. (1995). From science to practice. Meta-analyses using individual patient data are needed.. JAMA.

[PDF_00500] Steinberg K. K., Smith S. J., Stroup D. F., Olkin I., Lee N. C., Williamson G. D., Thacker S. B. (1997). Comparison of effect estimates from a meta-analysis of summary data from published studies and from a meta-analysis using individual patient data for ovarian cancer studies.. Am J Epidemiol.

[PDF_00504] Stewart L. A., Parmar M. K. (1993). Meta-analysis of the literature or of individual patient data: is there a difference?. Lancet.

[PDF_00506] Trallori G., Messori A., Scuffi C., Bardazzi G., Silvano R., d'Albasio G., Pacini F. (1995). 5-Aminosalicylic acid enemas to maintain remission in left-sided ulcerative colitis: a meta- and economic analysis.. J Clin Gastroenterol.

